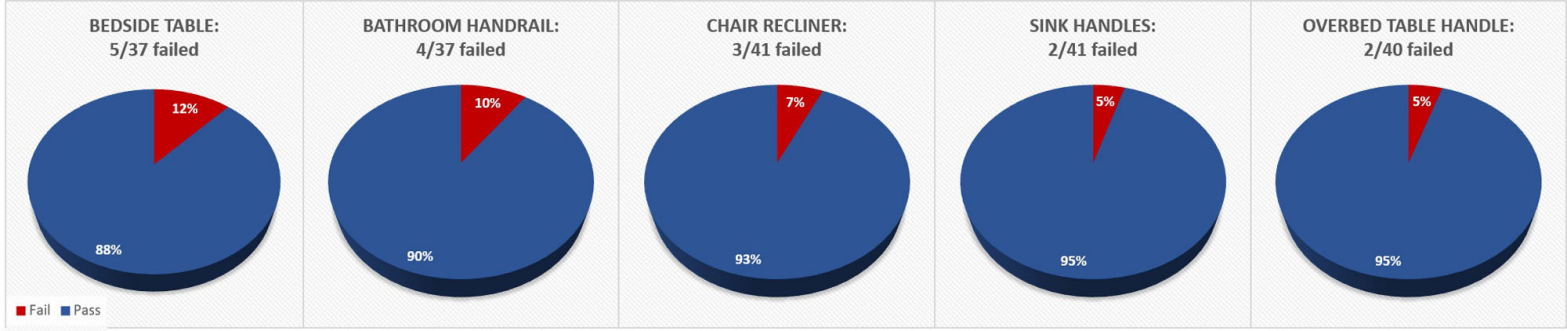# Improving Cleaning Validation Utilizing Adenosine Triphosphate Technology

**DOI:** 10.1017/ash.2024.239

**Published:** 2024-09-16

**Authors:** Mehvish Siddiqui, Rosemary Garcia, Rosa Lozano

**Affiliations:** Methodist Specialty and Transplant Hospital; Methodist Healthcare System

## Abstract

**Background:** Thorough cleaning and disinfection of high-touch surface areas in hospital inpatient rooms remain vital parts of effective strategies in reducing hospital-acquired infections (HAIs). Currently, Methodist Specialty & Transplant Hospital (MHST) inconsistently utilizes fluorescent marking for terminal cleaning validation. Without quantitative results, it’s difficult to measure the effectiveness of cleaning. To ensure MHST is maintaining a safe and clean environment for patients & staff, MHST implemented a comprehensive cleaning verification program to include adenosine triphosphate (ATP) technology. We aimed to establish the program with baseline readings, and an overall weekly passing score of 95% for all tested inpatient rooms. **Methods:** To achieve sustained improvement, we needed to monitor, educate, and have periodic performance feedback to individuals and stakeholders. Key stakeholders (IP, EVS, Operations Leadership, Nursing Leadership representative) were identified, and a weekly meeting was established to discuss the planning and implementation of the ATP program. Some key actions included: standardization of brand of luminometer- device to measure ATP for microbial contamination; establishment of 16 high surface touch points to be tested; partnership with IT to create a database & dashboard for ATP results & data analysis; training of ATP device to all personnel who will be utilizing ATP device; establishment of a threshold for a “pass” clean (relative light unit [RLU] less than or equal to 45). Summary of **Results:** After baseline testing, the average weekly pass score met goal at 95 percent for all tested rooms. The bedside table located on the 2W floor was the location that failed the most (3 instances). **Conclusions:** Our program implementation project aimed to improve terminal cleaning validation utilizing ATP technology in inpatient rooms, was successfully implemented. Equipped with quantitative results, the MHST team, was able to verify cleaning quickly and efficiently without any confusion, as it may have been with the previous verification method of fluorescent marking. The partnership between Infection Prevention (IP) & Environmental Services (EVS) was crucial in the implementation of this process improvement- from participating in training together to understanding and sharing ATP pass/fail score data.

**Figure:**
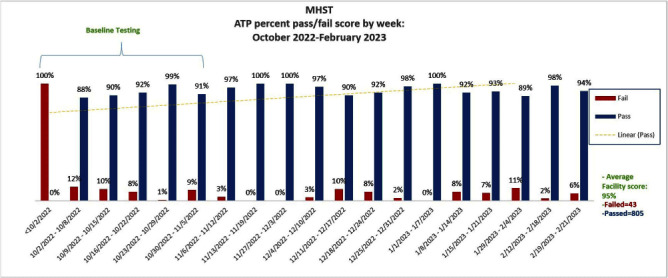

**Figure:**